# Cellular Effects of Everolimus and Sirolimus on Podocytes

**DOI:** 10.1371/journal.pone.0080340

**Published:** 2013-11-15

**Authors:** Sandra Müller-Krebs, Lena Weber, Julia Tsobaneli, Lars P. Kihm, Jochen Reiser, Martin Zeier, Vedat Schwenger

**Affiliations:** 1 Department of Nephrology, University of Heidelberg, Heidelberg, Germany; 2 Department of Medicine, Rush University Medical Center, Chicago, Illinois, United States of America; Fondazione IRCCS Ospedale Maggiore Policlinico & Fondazione D’Amico per la Ricerca sulle Malattie Renali, Italy

## Abstract

Everolimus (EVL) and Sirolimus (SRL) are potent immunosuppressant agents belonging to the group of mammalian target of rapamycin (mTOR) inhibitors used to prevent transplant rejection. However, some patients develop proteinuria following a switch from a calcineurin inhibitor regimen to mTOR inhibitors. Whether different mTOR inhibitors show similar effects on podocytes is still unknown. To analyze this, human podocytes were incubated with different doses of EVL and SRL. After incubation with EVL or SRL, podocytes revealed a reduced expression of total mTOR. Phosphorylation of p70S6K and Akt was diminished, whereas pAkt expression was more reduced in the SRL group. In both groups actin cytoskeletal reorganization was increased. Synaptopodin and podocin expression was reduced as well as nephrin protein, particularly in the SRL group. NFκB activation and IL-6 levels were lower in EVL and SRL, and even lower in SRL. Apoptosis was more increased in SRL than in the EVL group. Our data suggests that mTOR inhibitors affect podocyte integrity with respect to podocyte proteins, cytoskeleton, inflammation, and apoptosis. Our study is the first to analyze both mTOR inhibitors, EVL and SRL, in parallel in podocytes. Partially, the impact of EVL and SRL on podocytes differs. Nevertheless, it still remains unclear whether these differences are of relevance regarding to proteinuria in transplant patients.

## Introduction

The mechanism of action of Everolimus (EVL) and Sirolimus (SRL) is based on the inhibition of mammalian target of rapamycin (mTOR), a multiprotein complex [1,2] that directly influences protein synthesis and cell cycle progression. Both, EVL and SRL are used in transplant therapy to prevent rejection. One advantage of mTOR inhibitors over calcineurin inhibitors (CNI) is that they do not induce an increase in blood pressure and only cause mild nephrotoxicity [3]. This is of clinical interest, because CNI-induced nephrotoxicity is one of the prominent side effects in kidney, as well as heart transplantations [4,5]. Ten years after organ transplantation, interstitial fibrosis and tubular atrophy leads to end-stage renal disease in up to 20% of all heart transplant patients [6,7]. Nevertheless, it has been reported that some patients treated with mTOR inhibitors suffer from acute rejection, delayed graft function (DGF), and proteinuria [8-11]. The proteinuria could arise from the reorganization and degradation process of the podocytes. Generally, proteinuria is caused by a remodelling of the glomerular filter apparatus, in particular through morphological and functional changes of the podocytes, such as cytoskeletal rearrangements and foot process effacement [12].

In this context, it is of special interest to determine whether podocyte damage is dose dependently altered by EVL and SRL and whether there are differences of the two agents. Although EVL and SRL are both known to cause proteinuria [3,13], a single substance analysis in parallel with regard to the podocytes has not been performed either *in vitro* or *in vivo*.

Therefore, we investigated the mechanisms of mTOR inhibitor-induced podocyte damage, and demonstrate the impact of EVL and SRL on mTOR signaling on human podocytes.

## Materials and Methods

### Cell Culture of Podocytes

Conditionally immortalized human podocyte cells AB8/13 were cultured as described elsewhere [14]. In brief, podocytes were maintained at 33°C (permissive conditions). To induce differentiation, podocytes were cultured at 37°C (non-permissive conditions). After 14 days under non-permissive conditions, the cells revealed an arborized shape and expressed synaptopodin and podocin, which was shown by immunofluorescence or western blot analysis. The passages between 11 and 20 were used for this. 

### Incubation of Podocytes with mTOR Inhibitors

For incubation experiments, podocytes were serum deprived in differentiation medium containing 0.5% FCS for 24 h. Afterwards, the cells were incubated for 48 h with EVL (Novartis Pharma AG, Basel, Switzerland) and SRL (Sigma-Aldrich, Taufkirchen, Germany) in starvation medium. All experiments were repeated at least three times in each indicated condition. Concentrations of EVL and SRL between 0‑100 nM were used as kinetics and of 20 nM for direct comparison. As negative controls, we used untreated control cells (=C_Ø_) and solvent ethanol control (=C). Puromycin aminonucleoside (PAN) at 30 µg/ml (Santa Cruz Biotechnology, Heidelberg, Germany) served as positive control with regard to podocyte damage [15-17].

### Cell Viability

The release of lactate dehydrogenase (LDH) after incubation with EVL and SRL or PAN was measured in cell culture supernatants and assayed immediately for LDH using a cytotoxicity detection kit (Roche, Mannheim, Germany). Measurements were performed in triplicate according to the manufacturer’s instructions. 

A background control (providing information about the LDH activity in the assay medium), a low control (spontaneous LDH release), and a high control with 1% Triton X-100 (maximum LDH release) were carried out as described before [18]. Cytotoxicity is given in percent.

### Immunofluorescence

For immunofluorescence analysis, podocytes were grown in duplicate on type I collagen‑coated glass coverslips (Sigma-Aldrich, Taufkirchen, Germany), fixed with either methanol or 3% paraformaldehyde (PFA) phosphate-buffered saline (PBS) and incubated with primary antibodies and appropriate fluorescently labeled secondary antibodies. For nuclear staining, cells were stained with 0.1 mg/ml Hoechst 33342 (Invitrogen, Karlsruhe, Germany). The following primary antibodies were used: anti-nephrin, anti-podocin, anti-synaptopodin, and anti-NFκB (Santa Cruz Biotechnology, Heidelberg, Germany), anti-cleaved caspase-3 (Asp175) (Cell Signaling Technology, Danvers, MA). Except for phalloidin (Sigma-Aldrich, Deisenhofen, Germany), which was directly conjugated to fluorescein isothiocyanate (FITC), antigen-antibody complexes were visualized with DyLight 488 and Cy3-conjugated secondary antibodies (Dianova, Hamburg, Germany). Negative control was performed by using PBS instead of primary antibody. To analyze actin cytoskeleton organization, the number of cells which revealed a cortical reorganization of the actin cytoskeleton was counted and the total cell number was set to 100%. To analyze NFκB activation, the nuclear signal of NFκB-activated cells was counted and the total cell number was set to 100%. 

Images were taken using a Nikon DS-Qi1Mc quantitative black- and- white charge-coupled device camera attached to a Nikon Eclipse 80i upright microscope (Nikon, Düsseldorf, Germany). The same contrast and intensity settings were applied to samples stained with identical antibodies [19,20].

### FACS Analysis

After experimental incubation, 1x10^6^ cells were collected non-enzymatically and washed in 1% FCS-PBS; for intracellular staining, they were fixed with 3% PFA for 30 min, permeabilized with 0.2% Triton X for 5 min and stained for 1 h at 4°C using a directly FITC labeled anti-synaptopodin antibody (Progen, Heidelberg, Germany) and the respective isotype control (FITC Mouse IgG1, BD Biosciences, Heidelberg, Germany). After final washing, cells were resuspended in PBS and 10^4^ cells were acquired using a FACS Calibur Cytometer (Becton-Dickinson). CellQuest Software 3.3 was used for the analysis.

### TUNEL

Apoptosis was analyzed using terminal deoxynucleotidyl transferase mediated dUTP nick end labeling (TUNEL) assay (In Situ Cell Death Detection Kit, Fluorescein, Roche, Mannheim, Germany) according to the manufacturer’s recommendations. Following fixation in 3% buffered PFA, cells were incubated with terminal deoxynucleotidyl transferase using FITC labeled nucleotides. Hoechst 33258 served as a nuclear counterstain (blue); TUNEL positive cells (green) in 15–20 randomly selected fields were counted out of the total number of cells.

### Western Blot Analysis

Podocytes were serum starved for 24 h, incubated, lysed, and the protein concentration was assessed using the Pierce BCA Protein Assay Kit (Thermo Scientific, Langenselbold, Germany). Lysate (5–40 µg) was separated by 8–12% sodium dodecyl sulfate-polyacrylamide gel electrophoresis and transferred to a nitrocellulose membrane. The following antibodies were used: anti-mTOR, anti-pAkt (Ser473) (Cell Signaling Technology, Danvers, MA), anti-p70S6K, anti-p-p70S6K, anti-podocin, anti-synaptopodin, anti-RhoA, anit-CDC42 (Santa Cruz Biotechnology, Heidelberg, Germany), anti-Rac1 (BD Biosciences, Heidelberg, Germany), and anti-nephrin (C-terminus, Abcam, Cambridge, UK). Equal loading was verified by the detection of α-tubulin (Sigma-Aldrich, Deisenhofen, Germany). Protein detection was performed after incubation with primary and peroxidase-conjugated secondary antibodies using a SuperSignal Pico detection kit (Pierce, Bonn, Germany) according to the manufacturer’s instructions. To compare and quantify levels of proteins we applied ImageJ software based analysis. The area under curve (AUC) of the specific signal was corrected for the AUC of the loading control.

### Enzyme-Linked Immunosorbent Assay (ELISA)

IL-6 ELISA was performed according to the manufacturer’s instructions (human IL-6 ELISA kit, Bioscience, Inc., San Diego, CA). In brief, cells were incubated and supernatants were collected and diluted as described before [20]. Measurement was carried out at 450 nm using a Multiskan FC ELISA reader (Thermo Scientific, Langenselbold, Germany).

### Migration Assay

Differentiated podocytes were seeded in type I collagen-coated six-well plates and pretreated with solvent control, 20 nM EVL and SRL, and PAN. Wounding was performed with a 200 µl tip that was cut longitudinally in the well. Live cell recordings were performed immediately after wounding (0 h) and 24 h after the scratch using the motor-controlled Nikon Eclipse TE2000-E inverted microscope system (Nikon, Düsseldorf, Germany). Analyses were performed with NIS Elements AR 2.30 (Nikon, Düsseldorf, Germany) by counting the number of cells that had migrated into the sized fields [21]. For the statistical analysis, the wounded area from each well was measured in duplicate at 6 random wound gap locations per frame recorded per experiment. 

### Statistical Analysis

All of the data is shown as the mean (standard deviation) of at least three independent experiments in each indicated condition. The Mann-Whitney U test was carried out to test statistical significance. The significance level was set to at least *P* < 0.05. The statistical analysis was performed by PC-Statistik (version 5.0; Hoffmann, Giessen, Germany) and GraphPad Prism (version 4.03; San Diego, CA).

## Results

### Cell Viability

In comparison to the control podocytes, the EVL and SRL group showed an increase in cytotoxicity. As expected, the PAN group revealed the highest levels of LDH release (36.4% ± 16.8%; *P* < 0.001). Interestingly, cells incubated with SRL exhibited higher values of cytotoxicity than those incubated with EVL (EVL: 5.20% ± 5.22%; SRL: 12.3% ± 7.90%, *P* < 0.01).

### Proteins of the MTOR Signaling Pathway

#### mTOR protein

On the molecular level, EVL and SRL inhibit the multiprotein complex mTOR, which is the main component of a signaling cascade linked to cell proliferation.

**Figure 1:**
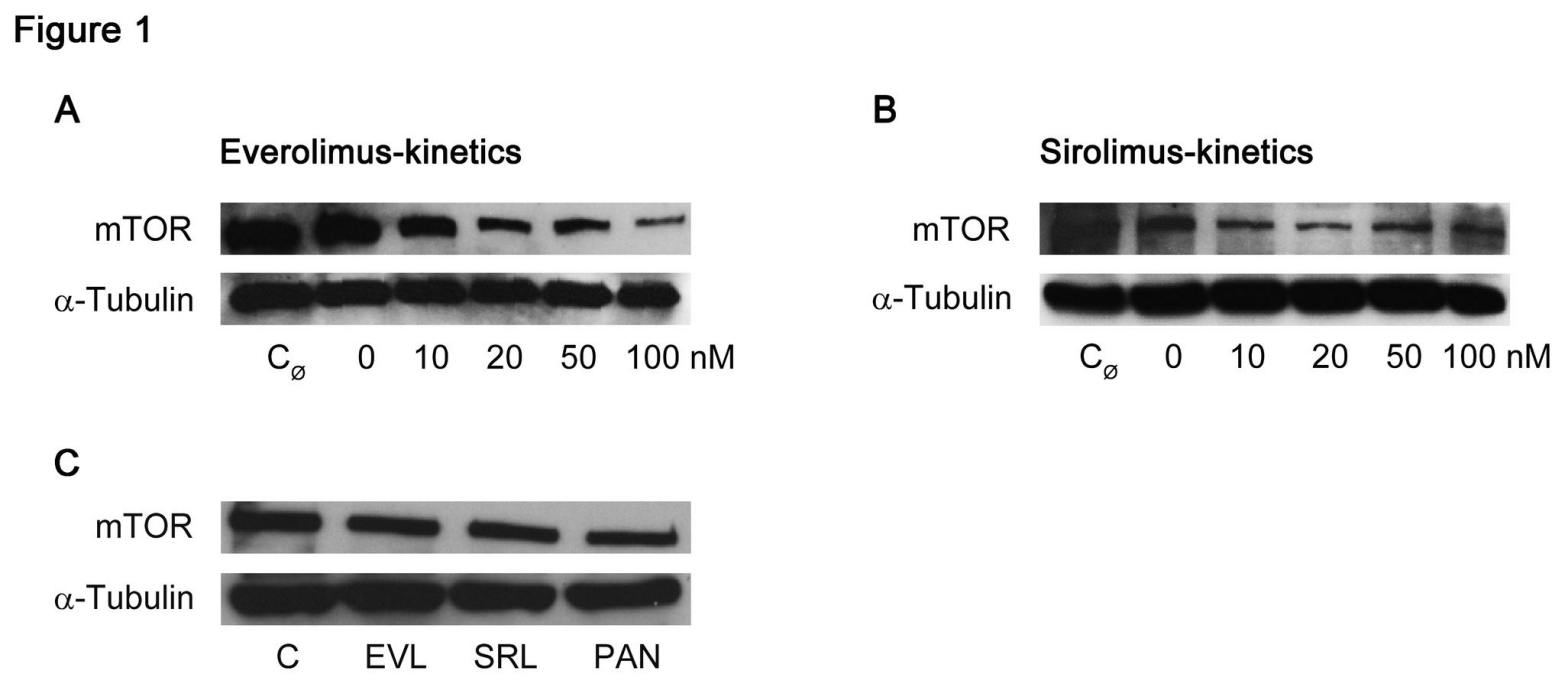
Decreased mTOR expression. Western blot analysis of podocytes showing decreased expression of total mTOR protein with increasing concentrations (0–100 nM) of EVL and SRL after 48 h of incubation, respectively (A, B); no differences in mTOR expression analyzing EVL and SRL (20 nM) simultaneously (C); α-tubulin served as loading control. mTOR = mammalian target of rapamycin; EVL = Everolimus; SRL = Sirolimus; C_Ø_ = untreated control; C = solvent ethanol control; PAN = puromycin aminonucleoside.

As shown by western blot analysis, podocytes treated with increasing concentrations (0–100 nM) of EVL or SRL for 48 h exhibited decreased expression of total mTOR protein. The signal of solvent free control (C_Ø_) and solvent control (C = 0 nM) did not reveal different intensities (Figure 1A, B). The expression of mTOR in the EVL and SRL groups was lower compared to control. PAN expression used as a positive control with regard to general podocyte damage was even lower. A comparison of EVL and SRL (20 nM) did not show any differences in total mTOR protein (Figure 1C). 

#### Downstream targets p70S6K and Akt

**Figure 2:**
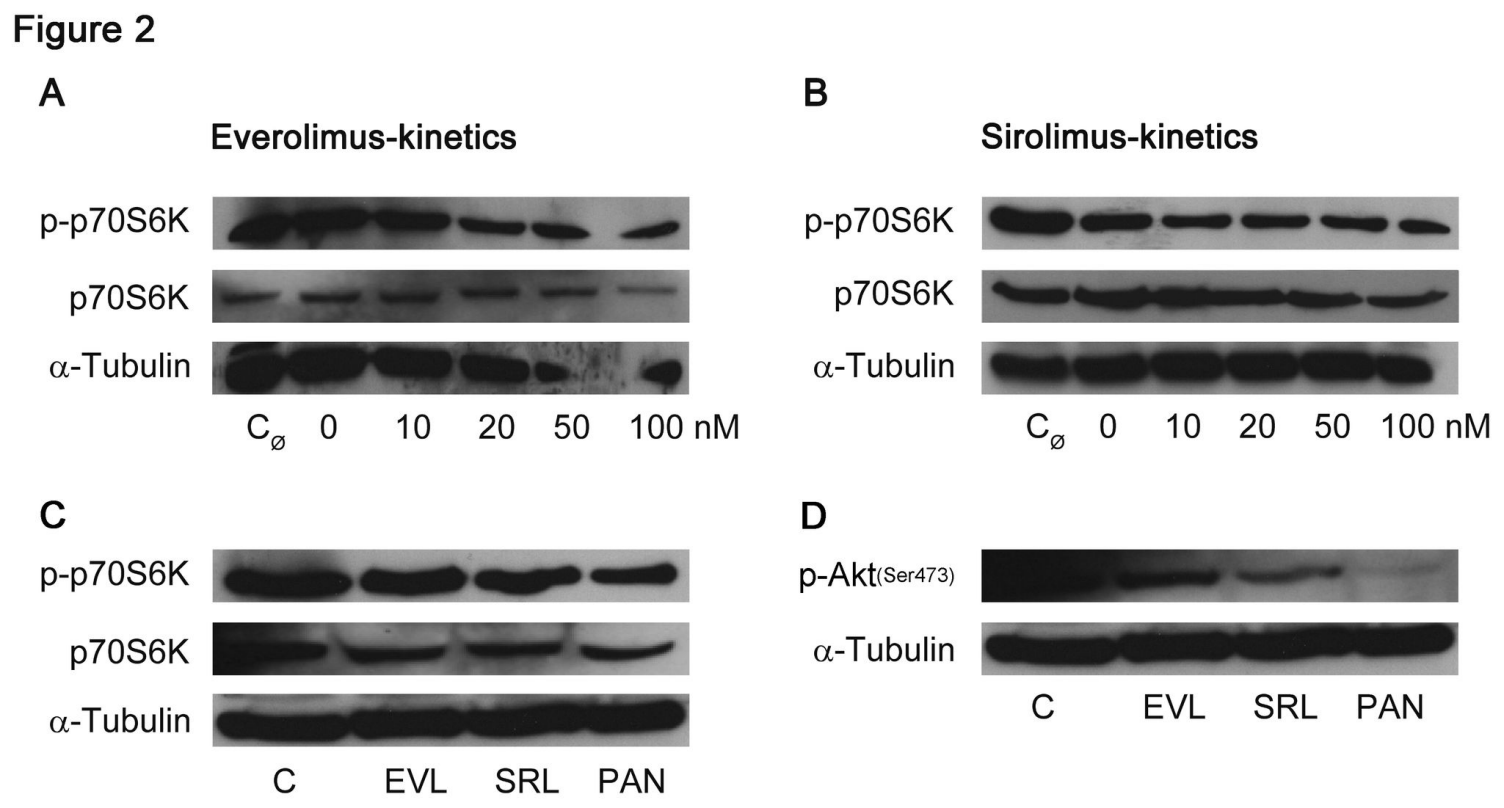
Reduction of mTOR downstream targets. Western blot analysis of podocytes showing a down-regulation of p-p70S6K, a downstream target of mTOR complex I, with a constant expression of total p70S6K with increasing concentrations (0-100 nM) of EVL and SRL after 48 h of incubation, respectively (A, B); no differences in p-p70S6K expression analyzing EVL and SRL (20 nM) in parallel (C); in contrast, expression of p-Akt (Ser473), a protein related to both mTOR complexes, was lower in SRL compared to EVL (D); α-tubulin served as loading control; analyses of p70S6K derived from different blots. p-p70S6K = phosphorylated p70S6 kinase; p70S6K = p70S6 kinase; p-Akt (Ser473) = phosphorylated Akt (Ser473); EVL = Everolimus; SRL = Sirolimus; C_Ø_ = untreated control; C = solvent ethanol control; PAN = puromycin aminonucleoside.

In the mTOR signaling cascade, the phosphorylation of proteins belonging to this pathway is of interest. Therefore, phosphorylation of the downstream target p70S6K that acts downstream to mTOR complex I was analyzed. We found diminished phosphorylation when cells were incubated with increasing concentrations of the immunosuppressant agents EVL and SRL, however, total p70S6K remained constant (Figure 2A, B; Figure S1A, A’, B, B’). EVL and SRL caused reduced phosphorylation of p70S6K versus control; however, no differences were observed in the agents themselves (Figure 2C; Figure S1C, C’).

In contrast, phosphorylation of Akt at Serin 473, which is another target in the mTOR pathway related to both, mTOR complex I and II, showed a higher reduction in SRL compared to EVL; almost no phosphorylation took place in PAN (Figure 2D¸ Figure S1D).

### Expression of Slit Diaphragm Associated Proteins

#### Nephrin

**Figure 3:**
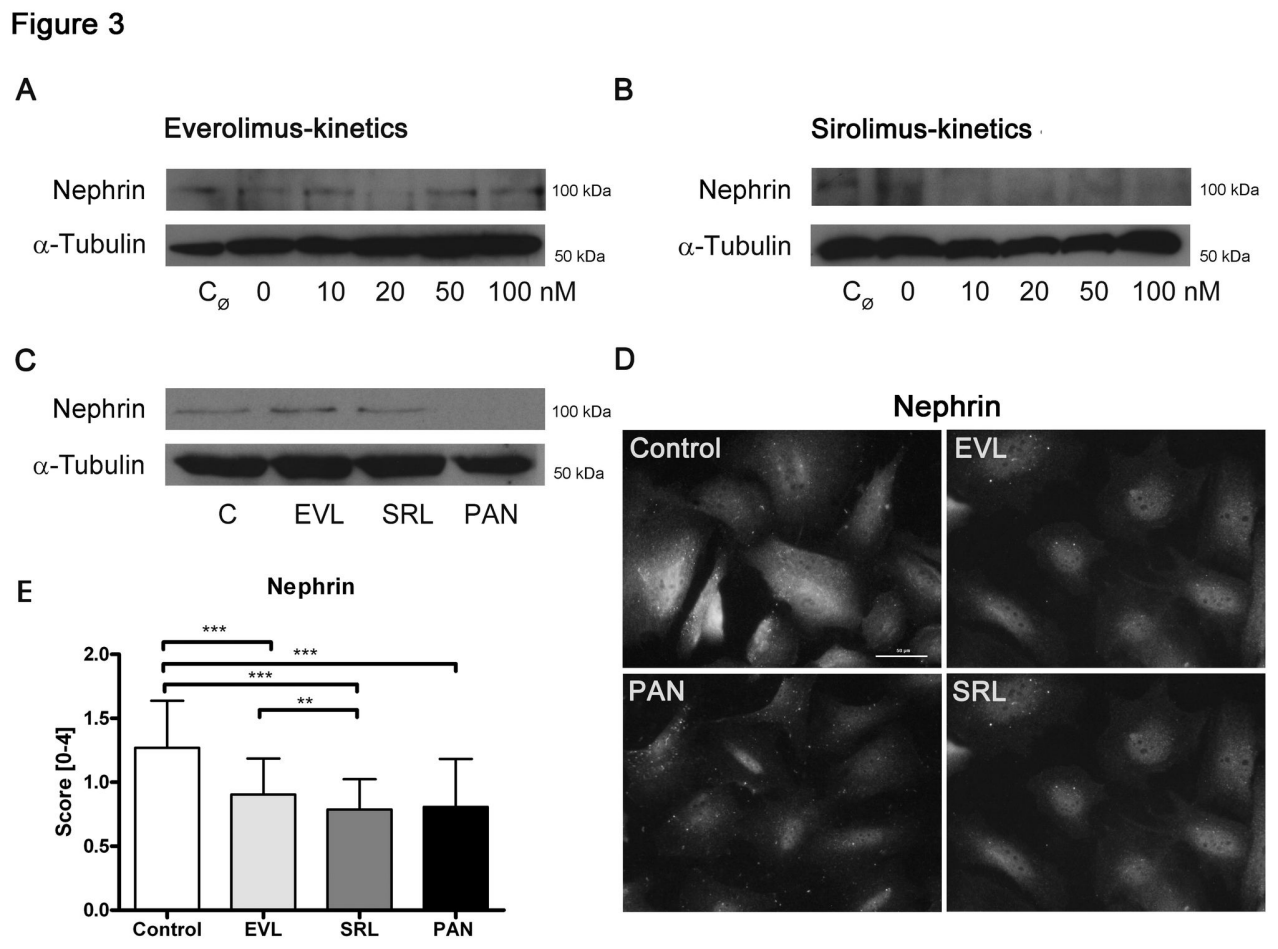
Decreased nephrin expression. Western blot analysis of podocytes showing a down-regulation of nephrin with increasing concentrations (0-100 nM) of EVL and SRL after 48 h of incubation, respectively (A, B); expression of nephrin in the simultaneous analysis (20 nM) was lower in SRL than in EVL (C); α-tubulin served as loading control. Immunofluorescence staining of nephrin (D) and analysis (E) revealed less nephrin protein in SRL compared to EVL. Magnification 400×, scale bar represents 50 µm. EVL = Everolimus; SRL = Sirolimus; C_Ø_ = untreated control; C, Control = solvent ethanol control; PAN = puromycin aminonucleoside. ***P < 0.001, **P < 0.01.

In a clinical study, it was observed that SRL application often leads to proteinuria. Moreover, it is known that reduction of the slit diaphragm associated protein nephrin leads to proteinuria *in vivo* [22,23]. Here, we observed that increasing concentrations (0–100 nM) of EVL or SRL resulted in decreased nephrin expression. The signal of solvent free control (C_Ø_) and solvent control (C = 0 nM) differed slightly (Figure 3A, B; Figure S2A, B). When analyzing EVL and SRL simultaneously, the western blot analysis showed that nephrin expression was lower in SRL than in EVL (Figure 3C; Figure S2C). These findings could be confirmed by immunofluorescence staining, which revealed less nephrin protein in SRL compared to EVL (Figure 3D, E). 

#### Podocin

**Figure 4:**
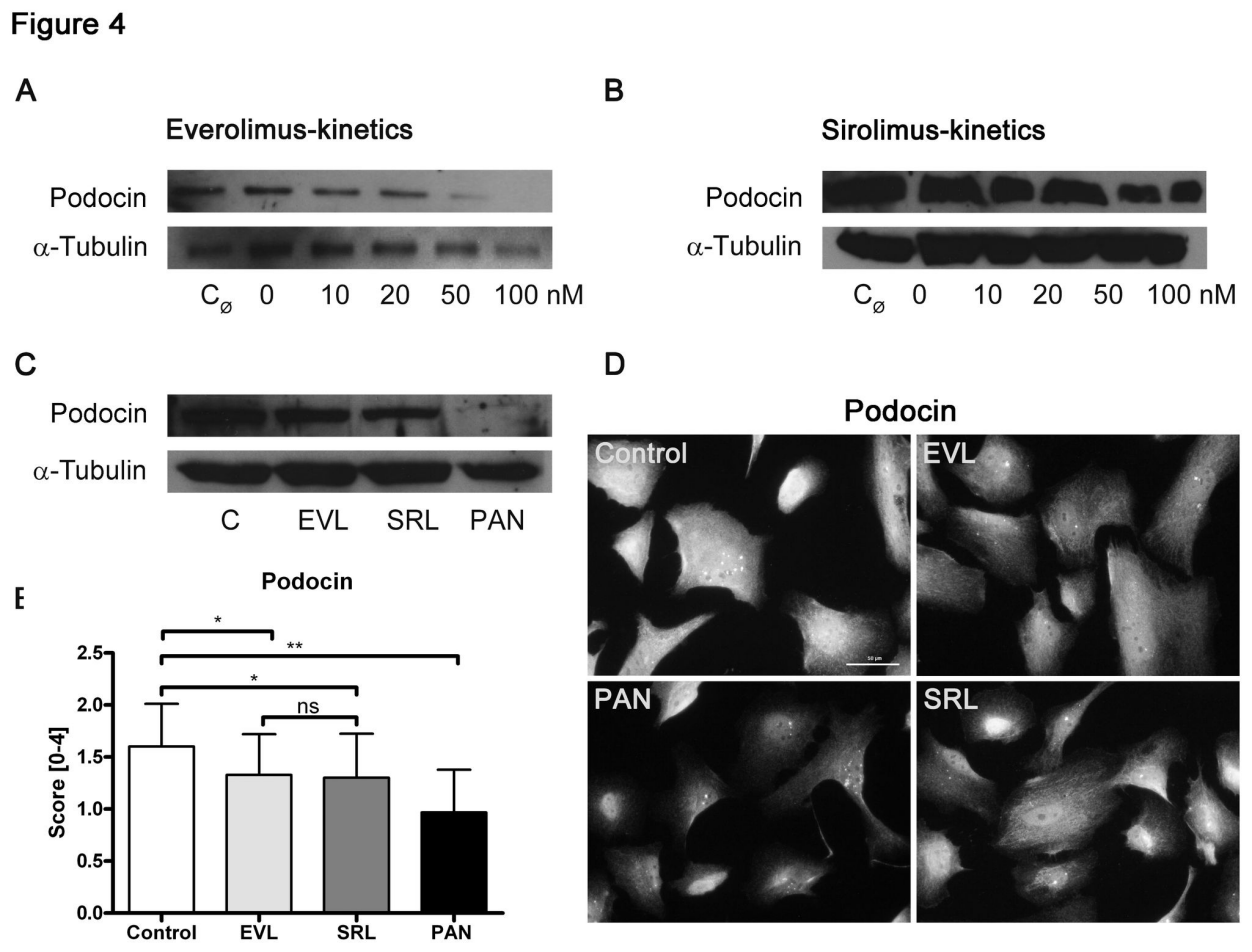
Reduction of podocin expression. Western blot analysis of podocytes showing a lower expression of podocin with increasing concentrations (0-100 nM) of EVL and SRL after 48 h of incubation, respectively (A, B); no differences in podocin expression analyzing EVL and SRL (20 nM) in parallel (C); α-tubulin served as loading control. Immunofluorescence staining of podocin (D) and analysis (E) did not show any changes in podocin protein in SRL compared to EVL. Magnification 400×, scale bar represents 50 µm. EVL = Everolimus; SRL = Sirolimus; C_Ø_ = untreated control; C, Control = solvent ethanol control; PAN = puromycin aminonucleoside; ns = not significant. **P < 0.01, *P < 0.05.

Mutations in the podocin gene NPHS2 cause steroid resistant nephrotic syndrome. Of note, this syndrome is accompanied by mild proteinuria, which has a delayed occurrence and even begins years after birth in some cases [24]. In our experiments, the impact of increasing concentrations (0–100 nM) of EVL or SRL on podocytes leads to lower podocin expression (Figure 4A, B). Podocin protein in the EVL and SRL groups was reduced in a similar way in terms of control; the effect of positive control PAN on deteriorated podocin expression was most prominent (Figure 4C). For the immunofluorescence analysis, comparable podocin staining in SRL and EVL could be observed (Figure 4D, E).

### Expression of Actin-Associated Proteins

#### Synaptopodin and RhoA

**Figure 5:**
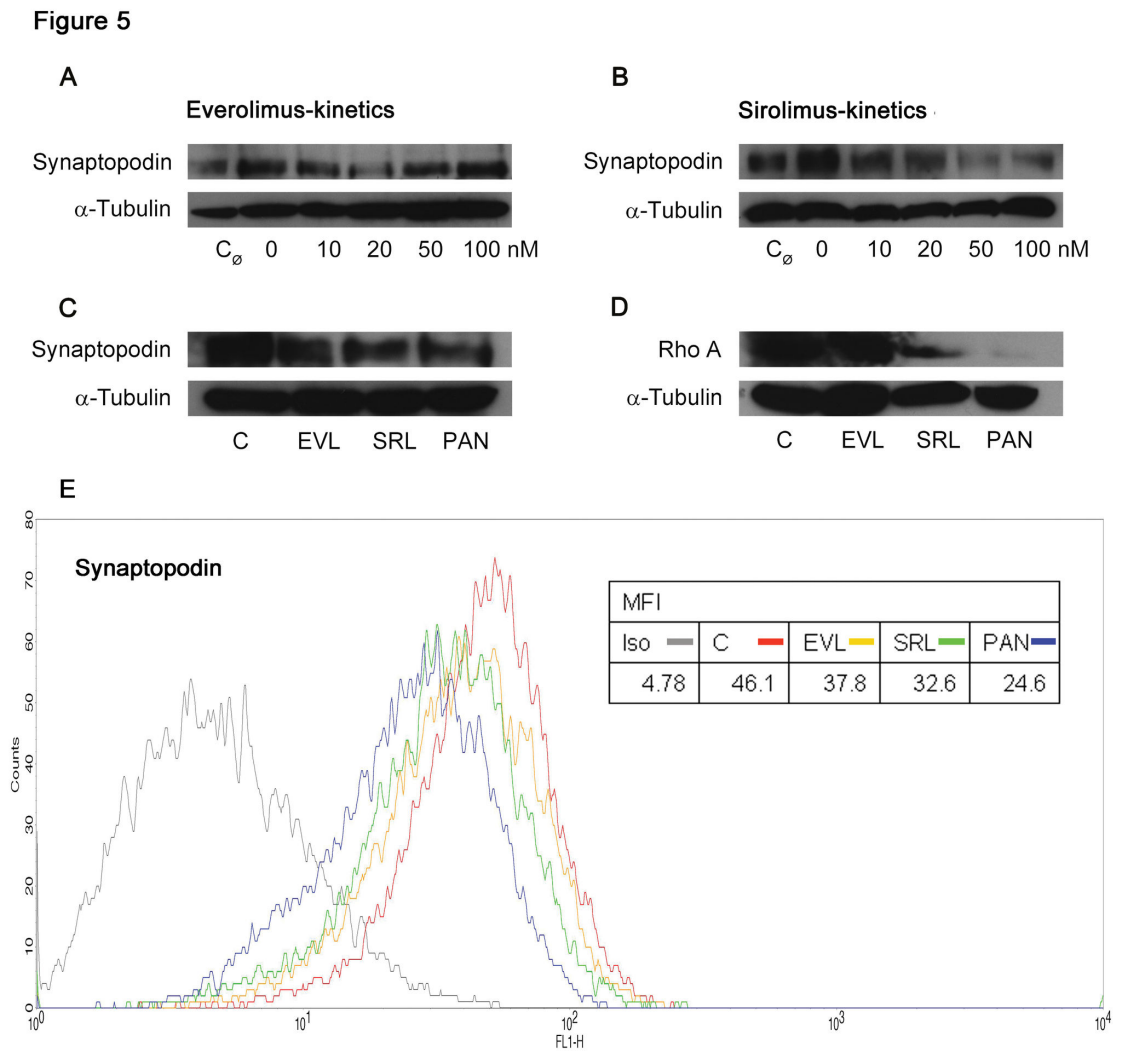
Decreased expression of synaptopodin and RhoA. Western blot analysis of podocytes showing a down-regulation of synaptopodin with increasing concentrations (0-100 nM) of EVL and SRL after 48 h of incubation, respectively (A, B); slightly reduced synaptopodin expression in western blot in a simultaneous analysis of EVL and SRL (20 nM) (C); diminished RhoA expression in the direct comparison in SRL (D); α-tubulin served as loading control. Representative flow cytometric analysis of synaptopodin expression on podocytes revealed reduced expression in SRL compared to EVL; the gray line represents isotype control, the red line represents C, the yellow line represents EVL, the green line represents SRL, and the blue line represents PAN (E). EVL = Everolimus; SRL = Sirolimus; C_Ø_ = untreated control; C, Control = solvent ethanol control; PAN = puromycin aminonucleoside; MFI = mean fluorescence intensity; Iso = isotype control.

Synaptopodin is an actin-associated protein of differentiated podocytes and part of the actin-based contractile apparatus in the foot processes [25]. It induces stress fibers by blocking the degradation of RhoA. In contrast to RhoA two further members of the Rho family, Rac1 and CDC42, are highly expressed, even when synaptopodin is missing [21].. In our western blot experiments, we found decreased synaptopodin expression when podocytes were incubated with increasing concentrations (0–100 nM) of EVL or SRL. The signal of solvent free control (C_Ø_) and solvent control (C = 0 nM) did not reveal any differences (Figure 5A, B). In a simultaneous analysis of EVL and SRL, the western blot analysis showed that synaptopodin expression was slightly lower in SRL than in EVL (Figure 5C; Figure S3). With regard to RhoA, we also found diminished expression of EVL and SRL, especially in the SRL group, meaning synaptopodin increases total RhoA expression (Figure 5D) whereas, as expected, the expression of the family members Rac1 and CDC42 remained unchanged in all groups (Figure S4A, B). We additionally analyzed synaptopodin expression by FACS analysis, which revealed less synaptopodin in SRL compared to EVL; the lowest expression of synaptopodin was present in PAN (Figure 5E). 

### Organization of the Actin Cytoskeleton

**Figure 6:**
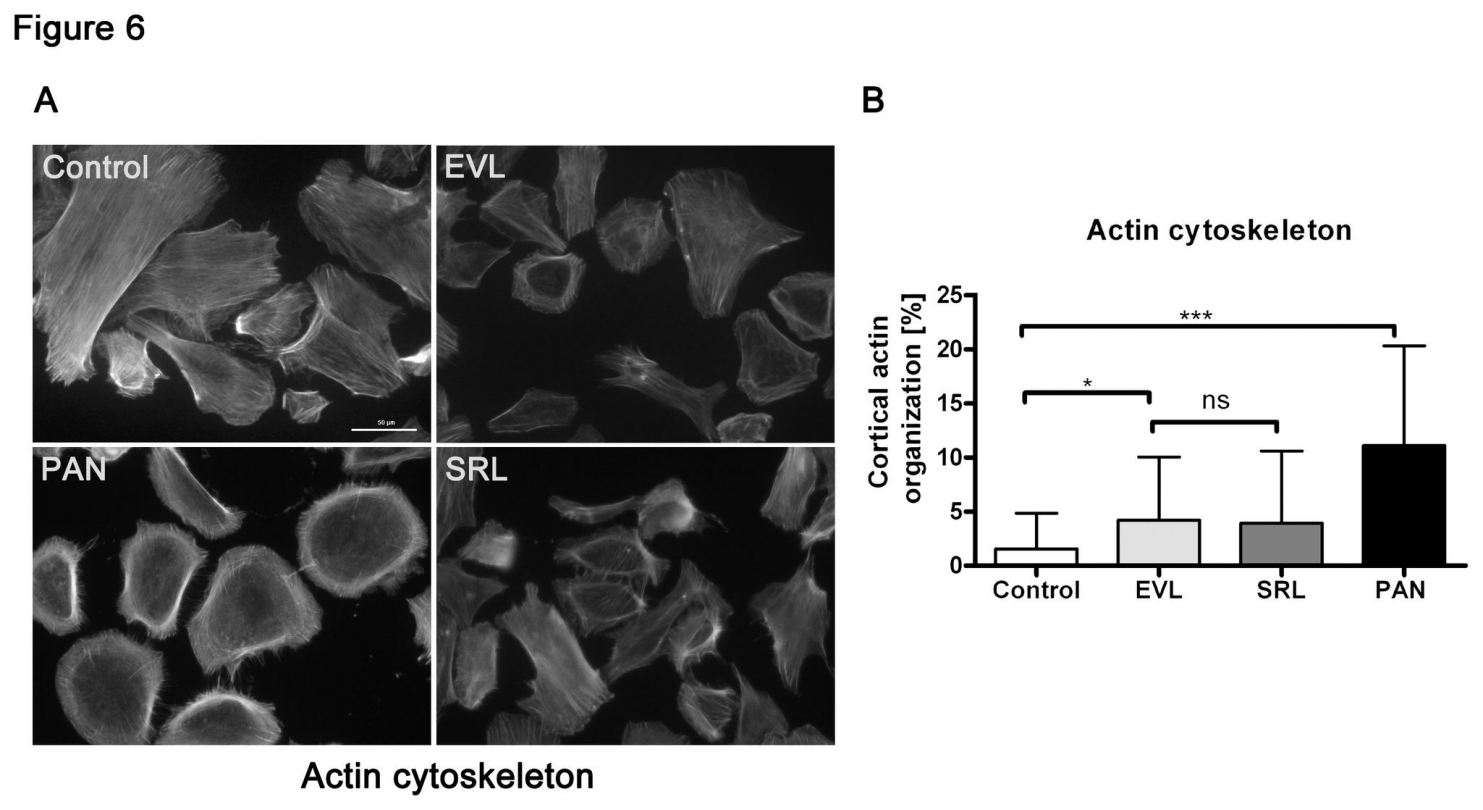
Reorganization of the actin cytoskeleton. Immunofluorescence staining of phalloidin to stain F-actin (A) and analysis (B) of podocytes showing a reorganization of the actin cytoskeleton after incubation with EVL and SRL for 48 h; no differences in the numbers of reorganized cells in the simultaneous analysis of EVL and SRL (20 nM) (B). Magnification 400×, scale bar represents 50 µm. EVL = Everolimus; SRL = Sirolimus; Control = solvent ethanol control; PAN = puromycin aminonucleoside; ns = not significant. ***P < 0.001, *P < 0.05.

The integrity of the cytoskeleton is important to preserve the functional morphology of podocytes. For this, we analyzed the actin cytoskeleton with respect to normal well-developed podocyte stress fibers or a reorganized cortical actin fiber phenotype using phalloidin staining. In our experiments, actin cytoskeletal reorganization was increased in both mTOR groups in comparison to control, but did not show any differences between EVL and SRL. The highest numbers of reorganized cells were found in the PAN group (Figure 6A, B).

### Migration Assay

**Figure 7:**
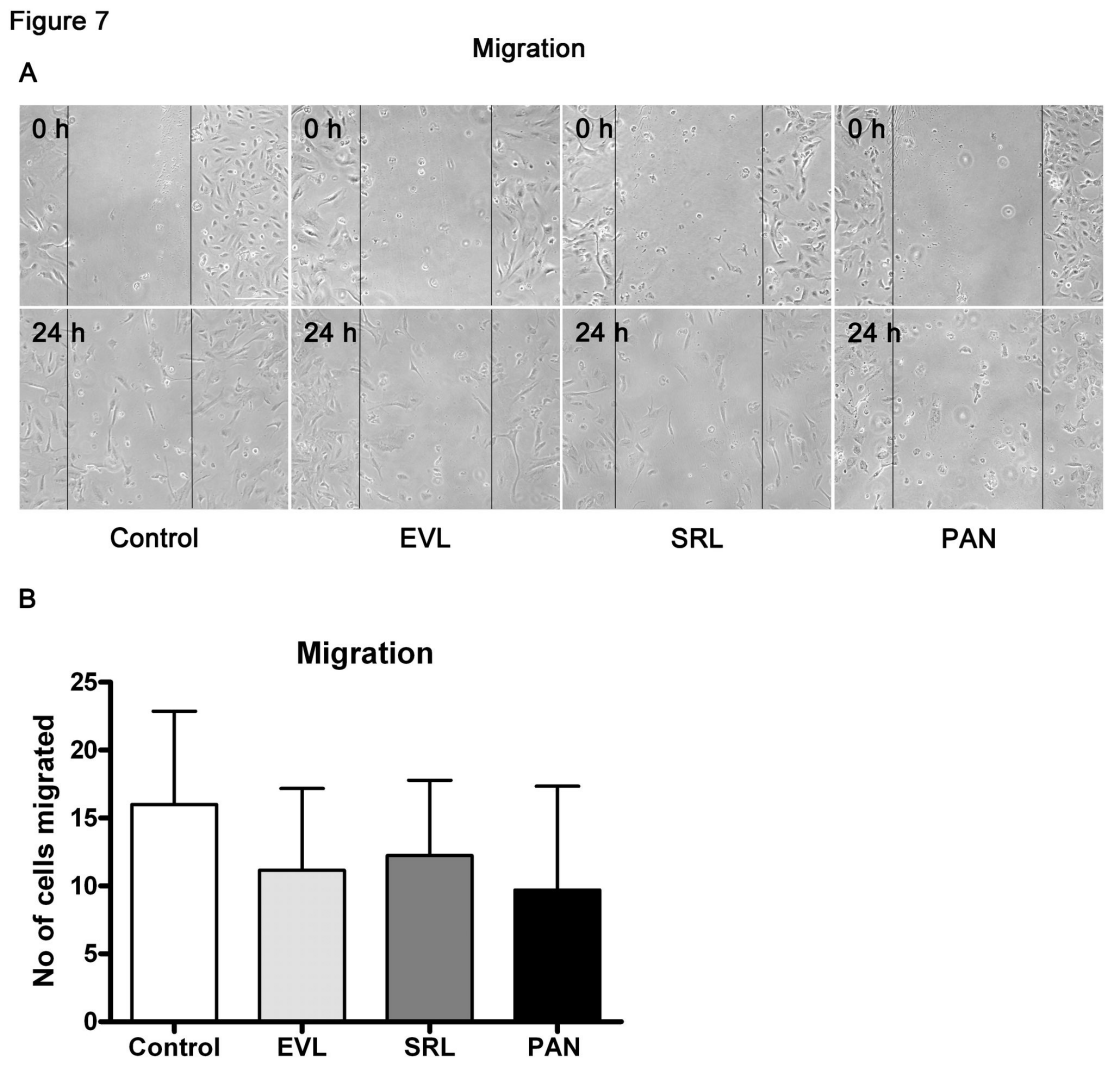
Reduced cell migration. Migration assay to investigate the effect of EVL and SRL (20 nM) on podocyte motility (A) after 48 h of incubation. The number of cells that migrated decreased in EVL and SRL in comparison to control (B); phase contrast microscopy. Magnification 100×, scale bar represents 200 µm. EVL = Everolimus; SRL = Sirolimus; Control = solvent ethanol control; PAN = puromycin aminonucleoside.

The dynamics of podocytes are associated with an intact actin-based contractile apparatus and physiological expression of synaptopodin [21]. To confirm that decreased synaptopodin expression also impairs directed podocyte migration, we performed a wound healing assay. After scratching, the wound healing was the best in the control group. In contrast, in the EVL and SRL groups, the number of migrated cells was reduced and the lowest motility was observed in PAN (Figure 7A, B).

### Inflammation

#### Regulation of NFκB

**Figure 8:**
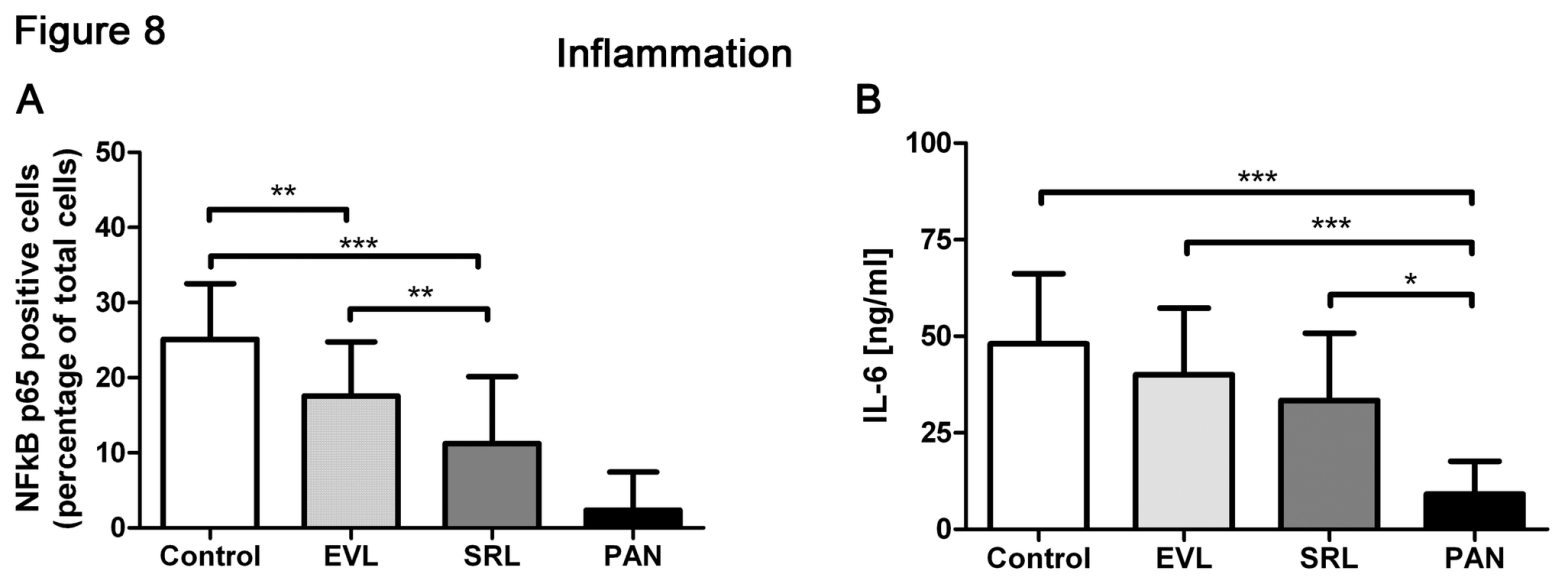
Decreased inflammation. Analysis of NFκB activation using immunofluorescence staining of NFκB p65 of podocytes after incubation with EVL and SRL for 48 h; differences can be found in the simultaneous analysis of EVL and SRL (20 nM); here, NFκB activation is lower in SRL than in EVL (A). ELISA analysis of IL-6 showed a reduced IL-6 release in the direct comparison of EVL and SRL (20 nM) with lower levels in the SRL group (B). EVL = Everolimus; SRL = Sirolimus; Control = solvent ethanol control; PAN = puromycin aminonucleoside. ***P < 0.001, **P < 0.01, *P < 0.05.

**Figure 9:**
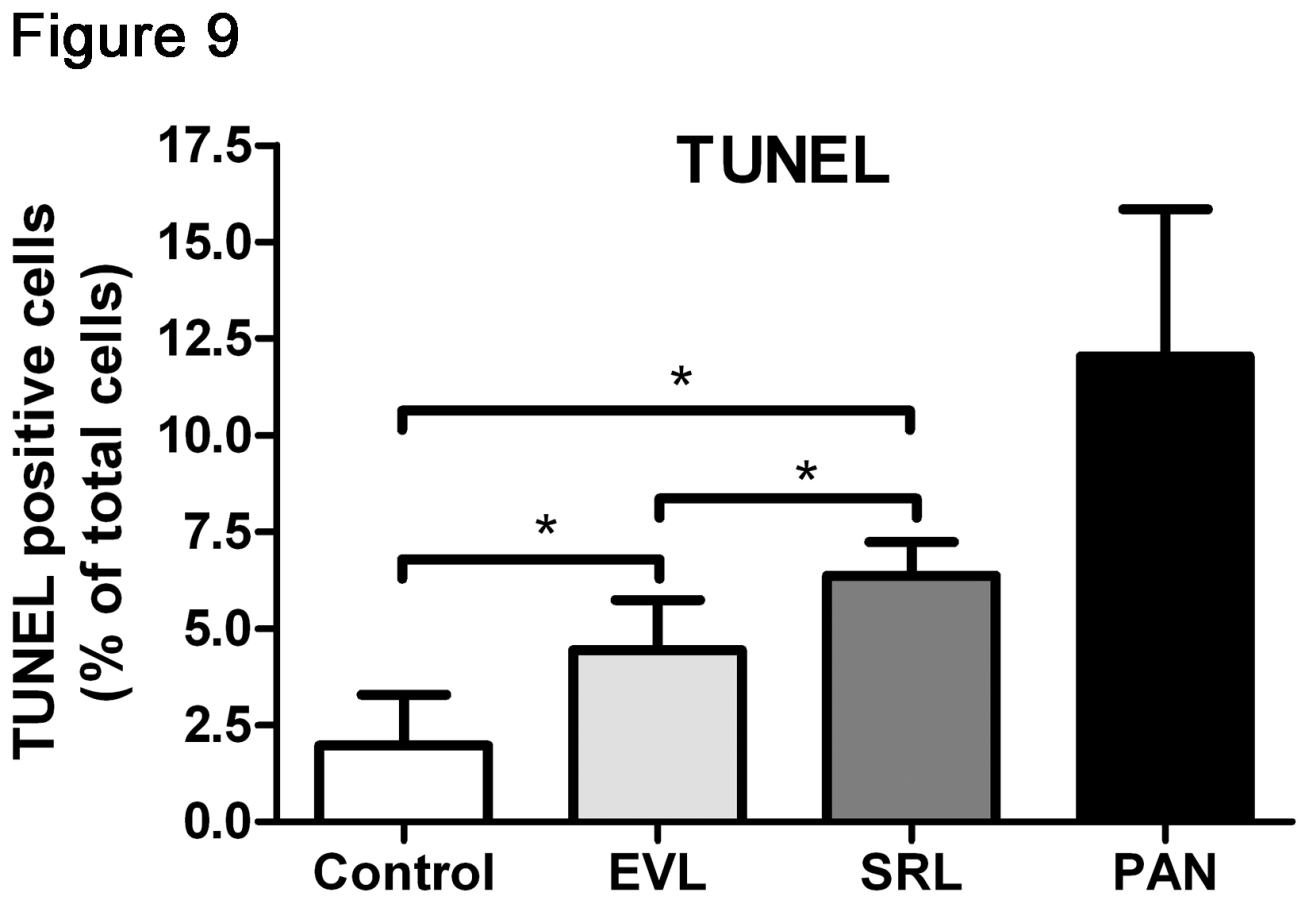
Increased apoptosis. TUNEL Assay of podocytes after incubation with EVL and SRL after 48 h of incubation; both mTOR inhibitor groups reveal higher numbers of apoptotic cells; differences can be found when analyzing EVL and SRL (20 nM) in parallel; more apoptotic cells were observed in the SRL group. EVL = Everolimus; SRL = Sirolimus; Control = solvent ethanol control; PAN = puromycin aminonucleoside; TUNEL = terminal deoxynucleotidyl transferase mediated dUTP nick end labelling. *P < 0.05.

NFκB activation is known to go along with an immune reaction resulting in an increased inflammatory status. Immunosuppressant agents should suppress this reaction. To test this, we analyzed the basal NFκB activation in control cells; in both mTOR inhibitor groups, NFκB activation was lower than in the control group and even more reduced in SRL than ELV. This could hint to a higher impact of SRL on the immune system than of EVL (Figure 8A, Figure S5). In the PAN group, virtually no NFκB activation was observable since PAN treated cells go directly into apoptosis (see Figure 9 below). 

#### Interleukin-6

NFκB activation was lower in both mTOR inhibitor groups as well as IL-6 excretion was reduced. Analysis of the IL-6 levels showed that IL-6 excretion was diminished especially in the SRL group compared to the EVL group. Basal IL-6 expression is reflected in the control group, whereas almost no IL-6 expression is detectable in the PAN group (Figure 8B).

### Apoptosis

To analyze apoptotic events, a TUNEL assay was performed, as well as immunofluorescence analysis of activated caspase-3. The number of TUNEL positive and activated caspase-3 positive cells was increased in both mTOR inhibitor groups as compared to control. Even more apoptotic events could be detected in SRL compared to EVL. As expected, the highest apoptosis was demonstrated in the PAN group (Figure 9, Figure S6).

## Discussion

To the best of our knowledge, our study is the first, which investigates the impact of EVL and SRL in parallel on a cell culture model of human podocytes. Our results shed new light on the differences in podocyte damage mediated dose dependently by either EVL or SRL. However, one has to remember that our analysis was performed in a podocyte cell culture system, where the natural microenvironment and thereby possible interactions are lacking.

When we incubated our cells for 48 h, we had to keep in mind that the half-life of EVL and SRL is different, it is 62 ± 16 h for SRL [26] and only 16-19 h for EVL [27]. Nevertheless, EVL with a shorter half-life than the 48 h incubation period rapidly diffuses into cells where it binds to its binding protein FKBP12 and is thus protected from degradation [28]. Since our approach has not been used before, we first had to find the appropriate dose to compare both reagents using concentration kinetics. Earlier studies conducted used doses of EVL or SRL ranging from 10 nm and 100 nm for 24 h to 120 h [29-31]. Given that concentrations of EVL and SRL of as low as 20 nM already result in strong mTOR inhibition, we decided to use a concentration of 20 nM to perform simultaneous analysis of EVL and SRL as the therapeutic dose, at least in renal transplant patients, correlates to 20 nM of mTOR immunosuppressant [32]. 

We incubated the cells with EVL and SRL and started by analyzing the mTOR pathway. Mechanistically, to act downstream of mTOR complex I, the target p70S6K needs to be sequentially activated by phosphorylation [33]. We found that this phosphorylation process was inhibited by EVL and SRL with no differences in the phosphorylation grade observable between the two agents; this demonstrates an efficient inhibition of mTOR complex I using EVL and SRL in podocytes. The situation is different regarding the phosphorylation of Akt. Akt is a protein related to both mTOR complexes. On the one hand, mTOR complex I can activate Akt indirectly following a negative feedback loop [34,35]; on the other hand, Akt can be directly phosphorylated via complex II. The simultaneous analysis of EVL and SRL demonstrated a stronger reduction of the phosphorylation of Akt in SRL treated podocytes, which hints to a higher effect of SRL on mTOR complex II and had been observed by Letavernier et al. after SRL incubation as well [30]. The question arose whether there are differences of EVL and SRL in podocytes besides those in the mTOR signaling pathway, particularly regarding to cytotoxicity, podocyte proteins, and apoptosis.

Our LDH assay revealed a high cytotoxicity in both mTOR inhibitor groups in comparison to control; interestingly, LDH release in the SRL group was higher than in the EVL group. At this point the literature is not concordant. Keller et al. and Letavernier et al. e.g. did not find cytotoxicity under mTOR regimen, in contrast to Vollenbrökers findings as well as to ours [29-31]. Our conclusion is therefore, that the toxic potential of SRL on podocytes might be more pronounced than that of EVL. To elucidate the toxic capacity on podocytes of both agents, the slit diaphragm associated protein nephrin was analyzed next. It is known that the reduction or absence of nephrin, e.g., due to mutations in the *NPHS1* gene, causes severe disorders, which ultimately result in massive proteinuria [24]. In our analyses, the expression of nephrin was markedly decreased in cells incubated with EVL and SRL, and more pronounced in SRL. This is in line with a recent study by Vollenbröker et al. in which a reduced nephrin expression was observable after SRL administration [31]. 

In contrast to nephrin, mutations in the podocin gene *NPHS2* are associated with less proteinuria, which occurs after years [24]. In accordance with these findings, we observed decreased podocin expression in both mTOR inhibitor groups, but no differences in the simultaneous analysis of EVL and SRL at 48 h. 

Another important podocyte specific protein is synaptopodin. Mechanistically, it stores podocyte integrity by blocking the degradation of RhoA, which induces stress fiber formation [21]. In our experiments, the expression of synaptopodin and RhoA was not as strong in the SRL group compared to EVL. Since a lack of synaptopodin is known to induce proteinuria [36], this finding might be clinically relevant as Letavernier et al. demonstrated in renal biopsies that synaptopodin staining was missing in focal lesions in patients undergoing SRL regimen [37]. Since we only analyzed podocyte markers as a possible source of glomerular proteinuria in this study it is of note that mTOR inhibitors also can interfere with the expression of proteins of the proximal tubule, resulting in decreased tubular mediated albumin endocytosis [38]. 

Our findings regarding the actin cytoskeleton revealed higher numbers of cells having a reorganized cytoskeleton in both mTOR inhibitor groups. Since migration is functionally linked to proteinuria and mechanistically linked to the actin cytoskeleton, we were interested in the migrational behavior of podocytes incubated with mTOR inhibitors. We found reduced migration in the mTOR inhibitor groups compared to control, but no differences of EVL and SRL in the parallel analysis. 

In general, the function of immunosuppressant agents is to reduce the immune system’s response. Therefore, it was not surprising that incubation with both, EVL and SRL, lead to reduced inflammatory response. Of note, inflammation was even lower in the SRL group. Since lower levels of inflammation are mostly advantageous, the impact of SRL on podocytes should be less harmful than that of EVL due to lower inflammation and potential reduced proteinuria. 

Another aspect of mTOR inhibitors in transplant patients is the problem of DGF, which particularly occurs after *de novo* SRL therapy [10]. Although the literature is inconsistent at that point, as some authors did not see any differences or even improvement using mTOR inhibitors with regard to DGF [8,9] it has been shown that in patients suffering from DGF, biopsy-proven increased tubular and podocyte apoptosis was demonstrated [39].

Due to the fact that we observed quite low inflammatory levels, the question came up of what happens to the cells after mTOR incubation. Do they become apoptotic? We performed a TUNEL assay as well as immunofluorescence staining of activated caspase-3 to determine this and found that apoptosis increased in both mTOR inhibitor groups and was even more elevated in the SRL group. Therefore, it can be concluded that these cells did not survive using the inflammatory pathway; instead, they went directly into apoptosis.

One possible result of apoptotic events in the glomerulus is protein excretion. Proteinuria is the result of various renal disorders in the absence or lack of synaptopodin [36]. The decrease or absence of proteinuria when using CNI seems to be due to the fact that CNI have a stabilizing effect on the actin cytoskeleton of podocytes, which is essential for the integrity of the glomerular filter [40]. When incubating podocytes with mTOR inhibitors, we did not only find reduced synaptopodin expression but also a reorganization of the actin cytoskeleton; this indicates potential risk to develop proteinuria *in vivo*, which has already been shown for the switch from CNI to SRL therapy [37].

The advantages and disadvantages of the different regimens have to be considered in the hypothetical application of our experimental data to the treatment of patients, as well as in analyzing the clinical studies carried out so far. On the one hand, CNI nephrotoxicity cannot be neglected; at the same time, however, CNI not only stabilize the actin cytoskeleton but also are more potent in the prevention of allograft rejection. On the other hand, when using mTOR inhibitors, podocyte damage resulting in proteinuria might occur. Currently, the mTOR inhibitors EVL and SRL are available. The focus of this study was a simultaneous analysis of the two. *De novo* SRL therapy has been reported to result in proteinuria and in focal segmental glomerulosclerosis at least to some degree. In these cases, diminished synaptopodin expression was observed [37].

Although an analysis of the mTOR inhibitors EVL and SRL in parallel in renal transplant therapy had not been done previously, there was already some evidence that SRL may be inferior to EVL with regard to toxicity in general. Rehm et al. reported that SRL-induced pneumonitis in kidney transplant patients was resolved after conversion to EVL [41]. In a recent study by Carvalho et al., a switch was made from an SRL to EVL regimen in renal transplant patients, but not vice versa in a crossover design; they did not find any differences between the drugs regarding e.g. serum creatinine or proteinuria [42]. In an animal study the combination of low dose CsA with either EVL or SRL with respect to nephrotoxicity was analyzed. The authors proclaim a reduced CsA nephrotoxicity in the combination of EVL with CsA [43].

## Conclusion

Our *in vitro* data point to differences in podocyte damage for EVL compared to SRL, in particular with regard to podocyte proteins and apoptosis. However, the clinical significance, especially with respect to the development of *de novo* proteinuria or delayed graft function, still has to be investigated.

## Supporting Information

Figure S1 **Reduction of mTOR downstream targets.** Quantification of western blot analysis of podocytes showing a down-regulation of p-p70S6K, (A, B), with a constant expression of total p70S6K (A’, B’) with increasing concentrations (0-100 nM) of EVL and SRL after 48 h of incubation, respectively; no differences in p-p70S6K expression (C) and p70S6K expression (C’) analyzing EVL and SRL (20 nM) in parallel; in contrast, expression of p-Akt (Ser473), was lower in SRL compared to EVL (D); α-tubulin served as loading control. p-p70S6K = phosphorylated p70S6 kinase; p70S6K = p70S6 kinase; p-Akt (Ser473) = phosphorylated Akt (Ser473); EVL = Everolimus; SRL = Sirolimus; C_Ø_ = untreated control; C = solvent ethanol control; PAN = puromycin aminonucleoside.
(TIF)

Figure S2 **Decreased nephrin expression.** Quantification of western blot analysis of podocytes showing a down-regulation of nephrin with increasing concentrations (0-100 nM) of EVL and SRL after 48 h of incubation, respectively (A, B); expression of nephrin in the simultaneous analysis (20 nM) was lower in SRL than in EVL (C); α-tubulin served as loading control. EVL = Everolimus; SRL = Sirolimus; C_Ø_ = untreated control; C, Control = solvent ethanol control; PAN = puromycin aminonucleoside.
(TIF)

Figure S3 **Decreased expression of synaptopodin.** Quantification of western blot analysis of podocytes showing a reduced synaptopodin expression in a simultaneous analysis of EVL and SRL (20 nM) after 48 h of incubation (C); α-tubulin served as loading control. EVL = Everolimus; SRL = Sirolimus; C, Control = solvent ethanol control; PAN = puromycin aminonucleoside.
(TIF)

Figure S4 **RhoA family members Rac1 and CDC42.** Western blot analysis of podocytes showing no mTOR inhibitor or PAN induced regulation of Rac1 and CDC42 after 48 h of incubation, respectively (A, B); α-tubulin served as loading control. PAN = puromycin aminonucleoside.
(TIF)

Figure S5 **Decreased inflammation.** Exemplary pictures of NFκB activation using immunofluorescence staining of NFκB p65 of podocytes after incubation with EVL and SRL for 48 h; differences can be found in the simultaneous analysis of EVL and SRL (20 nM); here, NFκB activation is lower in SRL than in EVL. Magnification 400×, scale bar represents 50 µm. EVL = Everolimus; SRL = Sirolimus; Control = solvent ethanol control.
(TIF)

Figure S6 **Increased apoptosis.** Exemplary pictures of caspase-3 activation using immunofluorescence staining of cleaved caspase-3 of podocytes after incubation with EVL and SRL for 48 h; differences can be found in the simultaneous analysis of EVL and SRL (20 nM); here, caspase-3 activation is higher in SRL than in EVL. Magnification 200×, scale bar represents 100 µm. EVL = Everolimus; SRL = Sirolimus; Control = solvent ethanol control.
(TIF)
