# Supplementary figures and images for: Cellular Effects of Everolimus and Sirolimus on Podocytes
Source: PLoS One. 2013 Nov 15;8(11):e80340. doi: 10.1371/journal.pone.0080340 (PMC3829970; doi:10.1371/journal.pone.0080340)

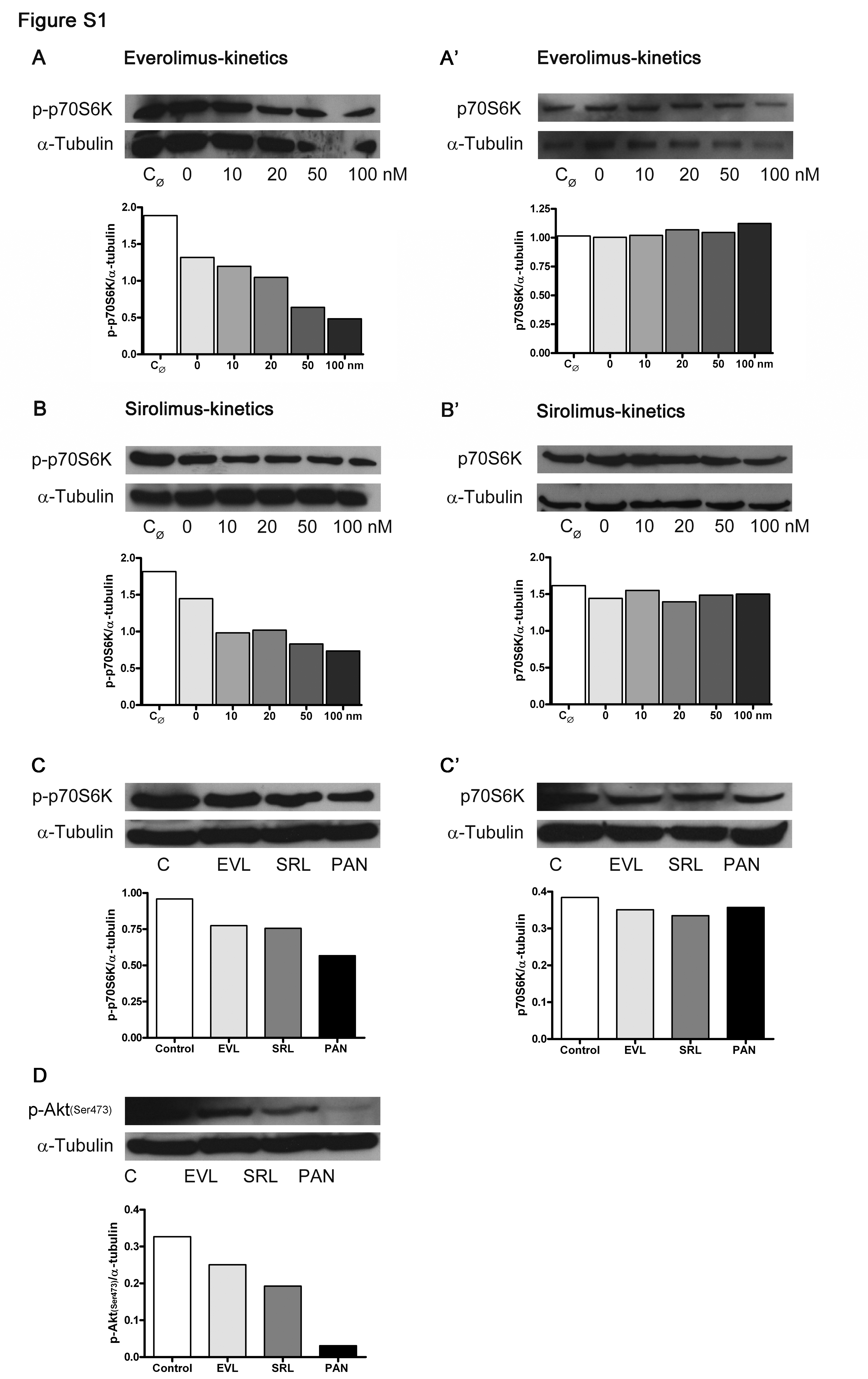

Supplement: Figure S1 — Reduction of mTOR downstream targets. Quantification of western blot analysis of podocytes showing a down-regulation of p-p70S6K, (A, B), with a constant expression of total p70S6K (A’, B’) with increasing concentrations (0-100 nM) of EVL and SRL after 48 h of incubation, respectively; no differences in p-p70S6K expression (C) and p70S6K expression (C’) analyzing EVL and SRL (20 nM) in parallel; in contrast, expression of p-Akt (Ser473), was lower in SRL compared to EVL (D); α-tubulin served as loading control. p-p70S6K = phosphorylated p70S6 kinase; p70S6K = p70S6 kinase; p-Akt (Ser473) = phosphorylated Akt (Ser473); EVL = Everolimus; SRL = Sirolimus; CØ = untreated control; C = solvent ethanol control; PAN = puromycin aminonucleoside. (TIF) [file pone.0080340.s001.tif]

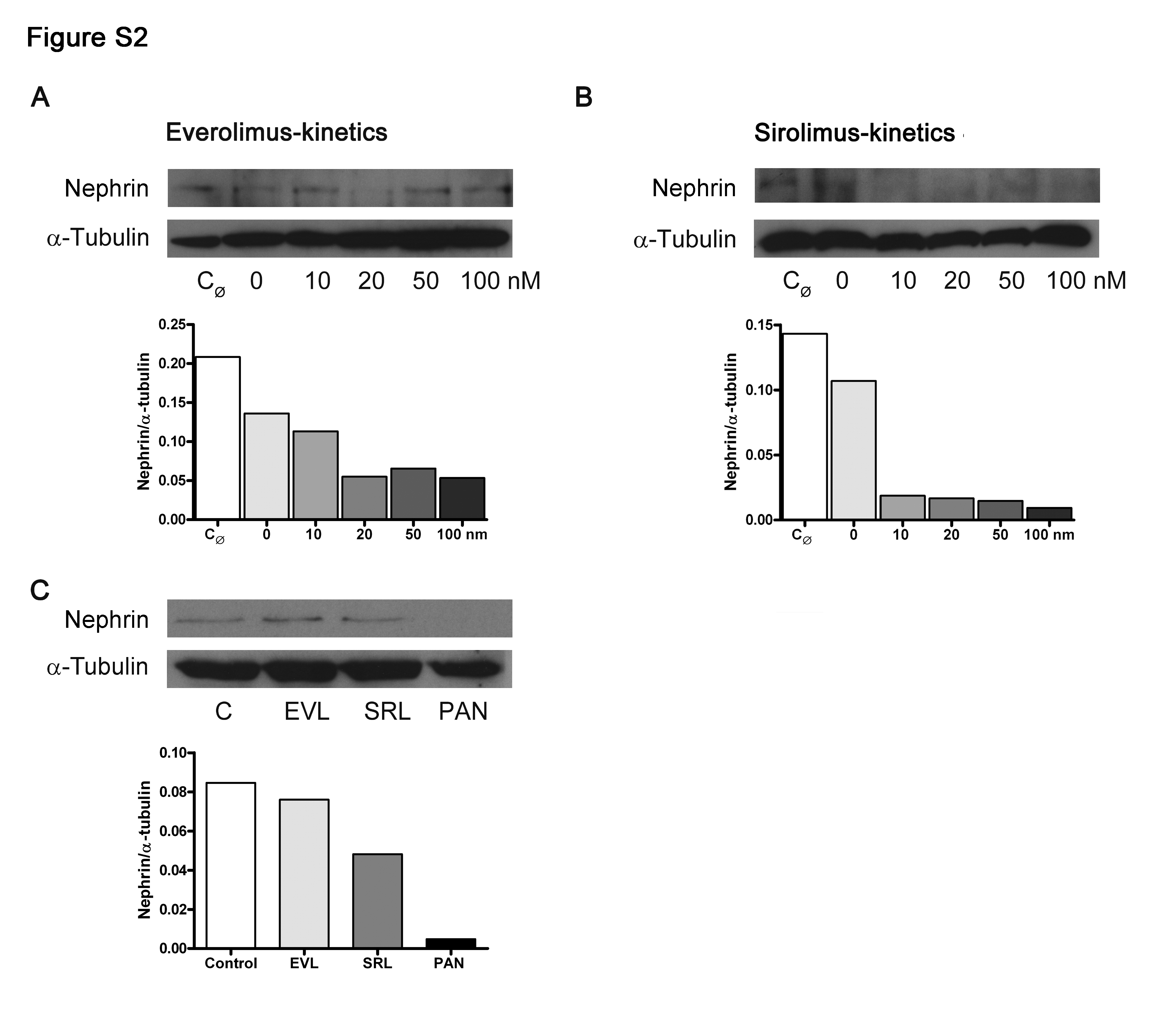

Supplement: Figure S2 — Decreased nephrin expression. Quantification of western blot analysis of podocytes showing a down-regulation of nephrin with increasing concentrations (0-100 nM) of EVL and SRL after 48 h of incubation, respectively (A, B); expression of nephrin in the simultaneous analysis (20 nM) was lower in SRL than in EVL (C); α-tubulin served as loading control. EVL = Everolimus; SRL = Sirolimus; CØ = untreated control; C, Control = solvent ethanol control; PAN = puromycin aminonucleoside. (TIF) [file pone.0080340.s002.tif]

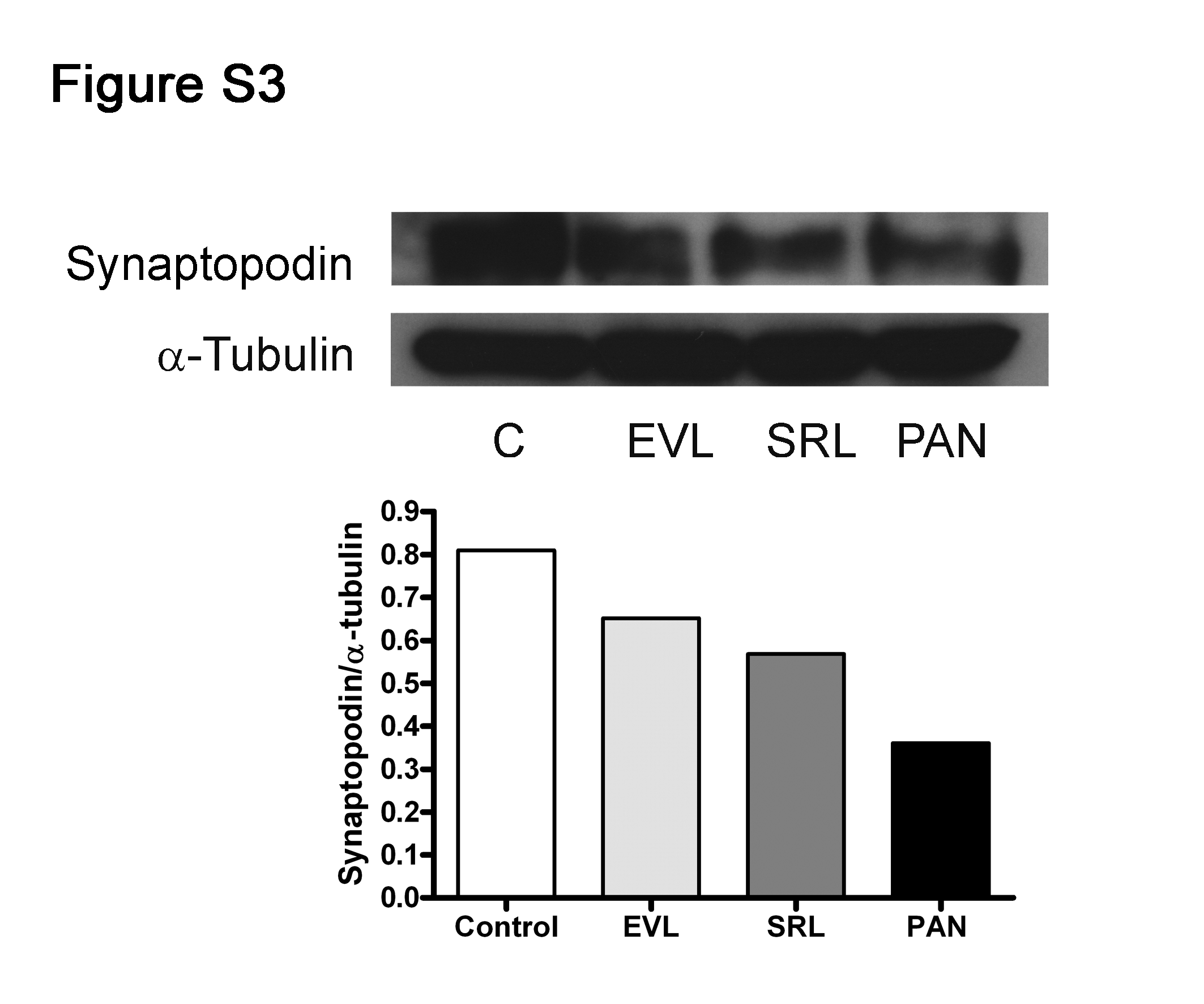

Supplement: Figure S3 — Decreased expression of synaptopodin. Quantification of western blot analysis of podocytes showing a reduced synaptopodin expression in a simultaneous analysis of EVL and SRL (20 nM) after 48 h of incubation (C); α-tubulin served as loading control. EVL = Everolimus; SRL = Sirolimus; C, Control = solvent ethanol control; PAN = puromycin aminonucleoside. (TIF) [file pone.0080340.s003.tif]

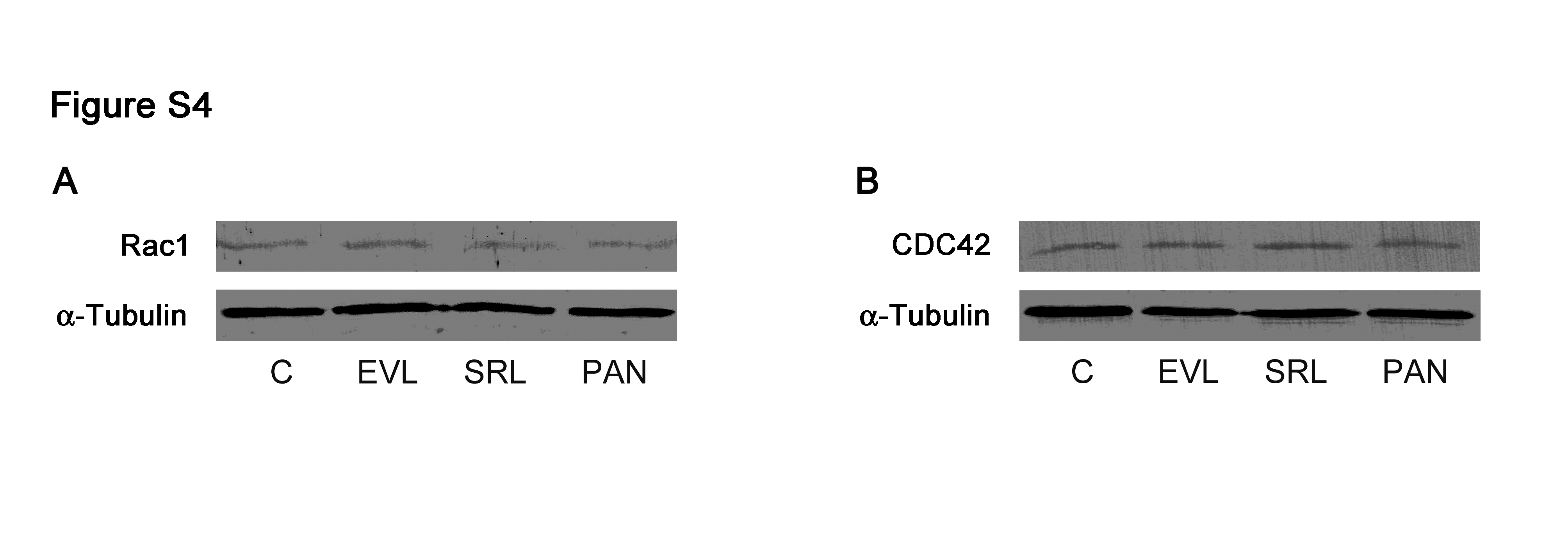

Supplement: Figure S4 — RhoA family members Rac1 and CDC42. Western blot analysis of podocytes showing no mTOR inhibitor or PAN induced regulation of Rac1 and CDC42 after 48 h of incubation, respectively (A, B); α-tubulin served as loading control. PAN = puromycin aminonucleoside. (TIF) [file pone.0080340.s004.tif]

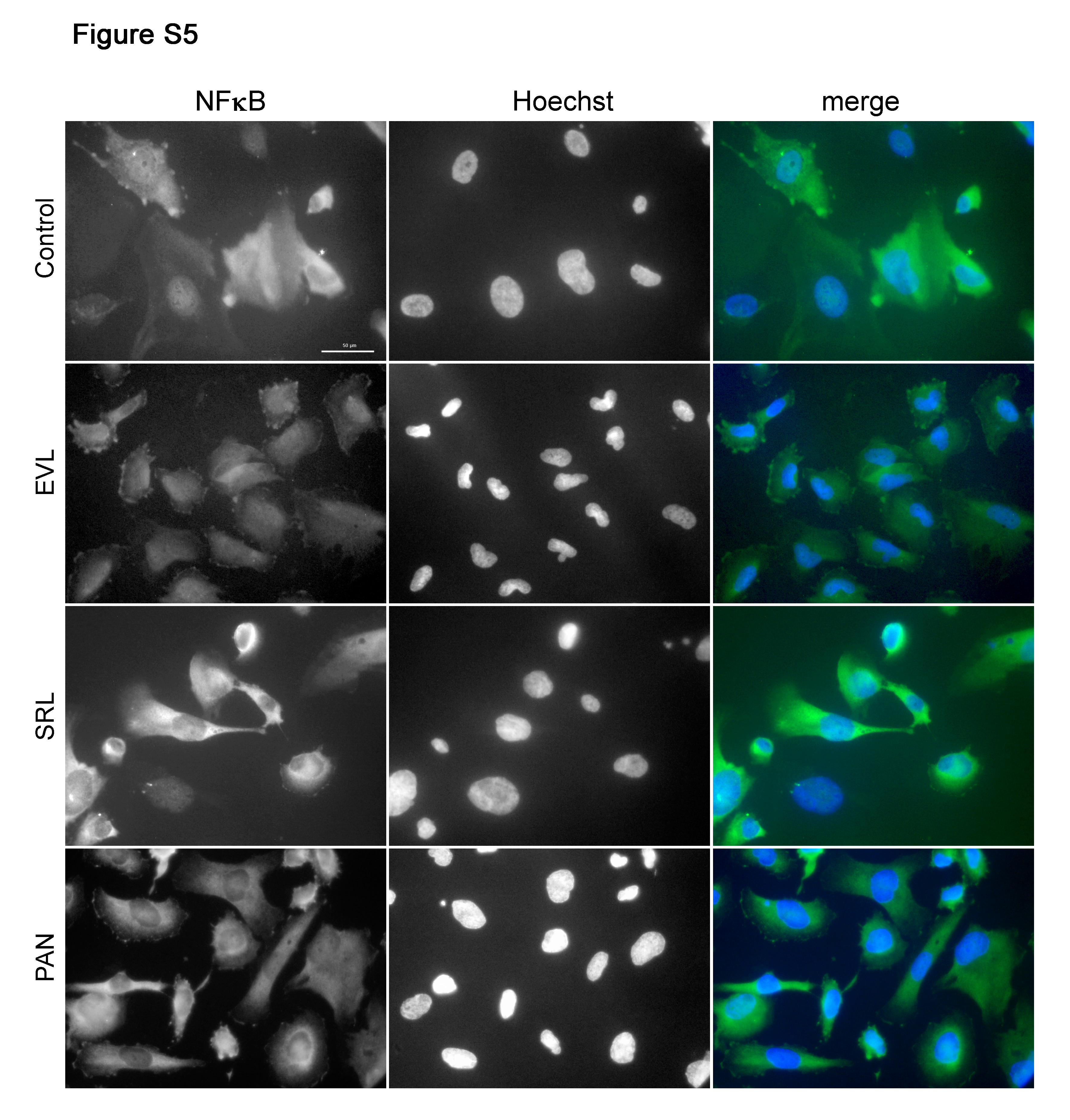

Supplement: Figure S5 — Decreased inflammation. Exemplary pictures of NFκB activation using immunofluorescence staining of NFκB p65 of podocytes after incubation with EVL and SRL for 48 h; differences can be found in the simultaneous analysis of EVL and SRL (20 nM); here, NFκB activation is lower in SRL than in EVL. Magnification 400×, scale bar represents 50 µm. EVL = Everolimus; SRL = Sirolimus; Control = solvent ethanol control. (TIF) [file pone.0080340.s005.tif]

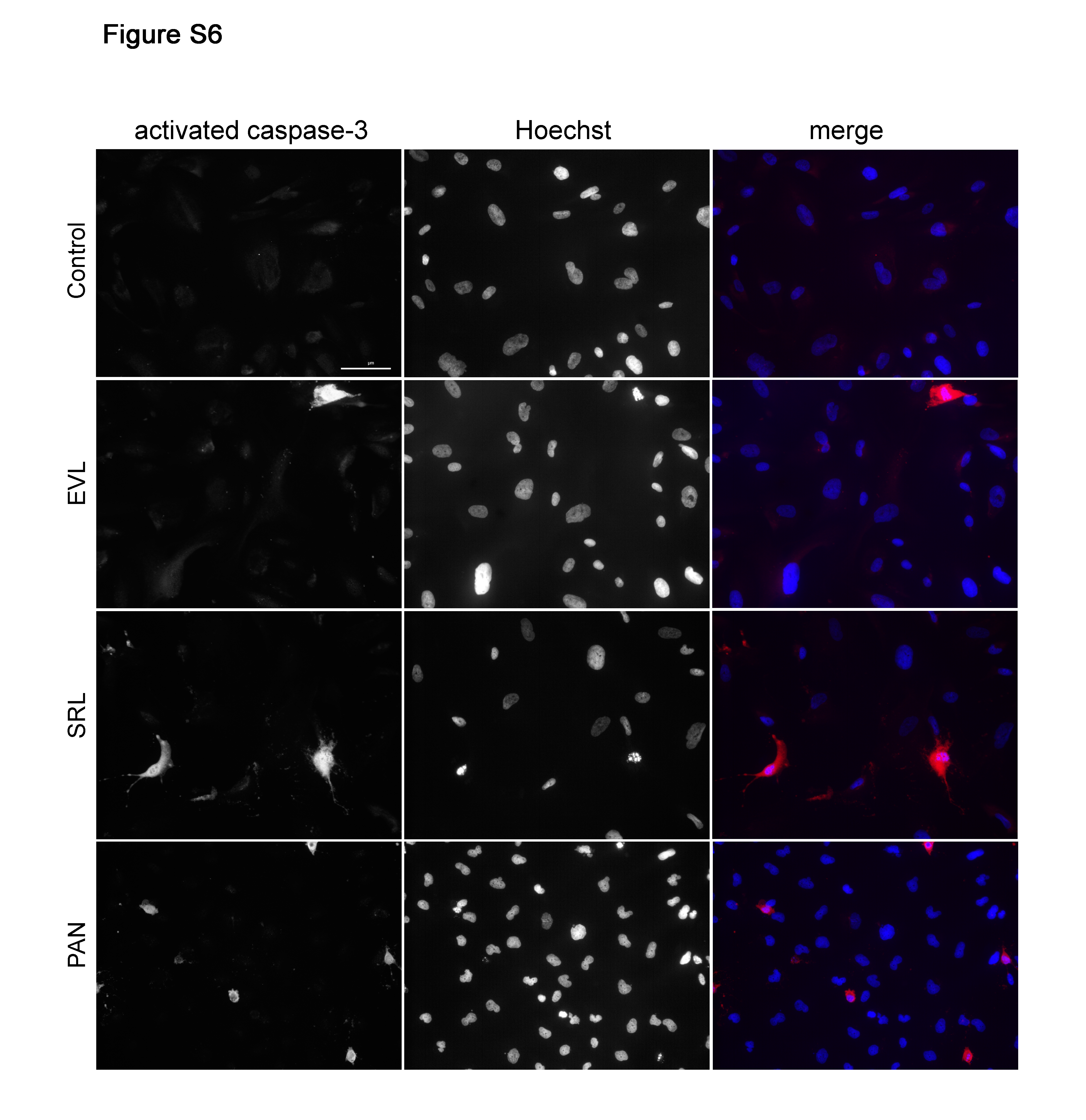

Supplement: Figure S6 — Increased apoptosis. Exemplary pictures of caspase-3 activation using immunofluorescence staining of cleaved caspase-3 of podocytes after incubation with EVL and SRL for 48 h; differences can be found in the simultaneous analysis of EVL and SRL (20 nM); here, caspase-3 activation is higher in SRL than in EVL. Magnification 200×, scale bar represents 100 µm. EVL = Everolimus; SRL = Sirolimus; Control = solvent ethanol control. (TIF) [file pone.0080340.s006.tif]
